# Causes of deaths in long-term care and hospice care facilities during the first year of COVID-19 pandemic: a snapshot of Italy during 2020

**DOI:** 10.1007/s40520-023-02426-7

**Published:** 2023-05-12

**Authors:** Maria Beatrice Zazzara, Giuseppe Ferdinando Colloca, Alice Maraschini, Andrea Bellieni, Sabrina Dispenza, Eleonora Meloni, Maria Adelaide Ricciotti, Italo Penco, Giada Minelli, Graziano Onder

**Affiliations:** 1 Fondazione Policlinico Universitario Agostino Gemelli IRCSS, Largo Agostino Gemelli 8, 00168 Rome, Italy; 2 Statistical Service, Istituto Superiore di Sanità, Viale Regina Elena, 299, 00161 Rome, Italy; 3 Fondazione Sanità e Ricerca, Via Alessandro Poerio, 100, 00152 Rome, Italy; 4 Università Cattolica del Sacro Cuore, Largo Francesco Vito, 1, 00168 Rome, Italy

**Keywords:** Palliative care, COVID-19, Hospice care, Long-term care, Nursing homes, Geriatrics

## Abstract

**Background:**

Older adults living in long-term care facilities (LTCF) have been severely affected by COVID-19. Hospice care (HC) facilities and palliative care are essential in treating patients dying from COVID-19. In Italy, little is known about the impact of COVID-19 on deaths in LTCF and the care provided in HC to COVID-19 patients.

**Aim:**

To assess overall and case-specific mortality in 2020 in LTC and HC facilities in comparison to the previous five years (2015–2019).

**Methods:**

We performed a descriptive study using data derived from the Italian national “Cause of Death” registry—managed by the Italian National Institute of Statistics—on deaths occurred in LTC and HC facilities during 2020 and the period 2015–2019.

**Results:**

Number of deaths significantly increased in 2020 compared with 2015–2019 in LTCF (83,062 deaths vs. 59,200) and slightly decreased in hospices (38,788 vs. 39,652). COVID-19 caused 12.5% of deaths in LTCF and only 2% in hospices. Other than COVID-19, in 2020, cancer accounted for 77% of all deaths that occurred in HC, while cardiovascular diseases (35.6%) and psychotic and behavioral disorders (10%) were the most common causes of death in LTCF. Overall, 22% of the excess mortality registered in Italy during 2020 is represented by the deaths that occurred in LTCF.

**Discussion and conclusion:**

LTCF were disproportionally affected by COVID-19, while the response to the pandemic in HC was limited. These data can help plan strategies to limit the impact of future epidemics and to better understand residential care response to COVID-19 epidemic.

**Supplementary Information:**

The online version contains supplementary material available at 10.1007/s40520-023-02426-7.

## Introduction

COVID-19 poses a direct threat to older adults, who are intrinsically more at risk of adverse outcomes [1–3], are usually affected by multiple chronic conditions, and experience geriatric syndromes, such as frailty and cognitive impairment [4, 5]. Italy, one of the countries with the oldest population worldwide [6], has registered an excess mortality of more than 100,000 deaths in 2020, the first year of the epidemic, and the highest case-fatality rate has been observed among older multi-morbid patients [7].

Older adults in long-term care facilities represent a population that has been particularly affected by COVID-19 due to the intrinsic vulnerability of the residents, who are often institutionalized because of the higher prevalence of multiple health conditions and disability [2, 8, 9]. Also, the restrictions set in place by health authorities to reduce the spread of the virus, such as social distancing and limited visiting hours with family members, have contributed to increasing social isolation, frailty, and incident adverse events [10]. In England, during the first weeks of the pandemic, an excess mortality rate was registered among care homes residents compared to community dwellers [11, 12]. An Italian national survey of 1,356 nursing homes, hosting a total of 100,806 residents, recorded 9,154 overall deaths during the first trimester of the pandemic, with a cumulative incidence of 0.7 per 100 residents among COVID-19 confirmed cases and 3.1 per 100 residents among flu‐like symptoms cases [13, 14]. The lack of personal protective equipment (PPE) and limited availability of COVID-19 testing have also contributed to the increased mortality [11–13]. To this date, in Italy, the exact impact of COVID-19 on overall deaths in long-term care facilities is unknown.

Hospice care facilities are designed to provide multidimensional care, aiming to reduce physical, psychological, social, and spiritual suffering [15, 16]. The relief of suffering, but also the support of complex decision-making, the management of clinical uncertainty, the provision of psychosocial and bereavement care, and the advocacy for patients and their families are critical features of palliative care and essential components of the response during global emergencies such as epidemics [17–19]. Two systematic reviews have highlighted how palliative care services in hospitals and primary healthcare are essential in response to COVID-19 [18, 19], and could play a crucial role in treating patients dying from COVID-19 [17]. A study conducted in Lombardy, Italy, underlined how palliative care in an emergency context could contribute to patient selection and triage through a multidisciplinary approach to discuss the best therapeutic strategy on a case-by-case basis [20]. Older frail multi-morbid adults with complex clinical and social needs could greatly benefit from a care plan that includes palliative management, thus avoiding unnecessary hospitalization or a disproportionate intensive clinical approach [21]. In October 2020, the Palliative Care Federation (FCP) published a joint document with the Italian Palliative Care Society (SICP) suggesting recommendations and guidelines to integrate palliative care into the national pandemic plan [22]. However, little is known about the care provided in hospice to patients suffering from COVID-19 at a country level.

With this work, we aimed to assess case-specific mortality in 2020 in long-term care and hospice care facilities compared to the previous five years (2015–2019), with a specific focus on COVID-19. We also aimed to evaluate excess mortality by cause in these settings.

## Methods

Deaths of the Italian resident population that occurred in 2015–2020 were analyzed. We compared different causes of death observed in 2020 in the Italian resident population to the ones observed in the previous five years (2015–2019), in terms of the mean number of casualties. Causes of death were derived from the national Cause of Death registry, managed by the Italian National Institute of Statistics, which collects copies of death certificates completed by the medical certifiers for all deaths occurring in Italy. All causes reported on the death certificate are classified according to the International Classification of Diseases, 10th Revision (ICD10) [23], using the semi-automated coding system Iris (www.iris-institute.org, accessed on 23 November 2022), which attributes ICD codes for approximately 80% of cases; the remaining 20% are reviewed by expert personnel. For each case, the underlying cause of death defined by the World Health Organization (WHO) as “the condition that initiated the train of morbid events leading to death” was selected. COVID-19 coding was performed by the latest recommendations by the WHO [24].

### Study settings

The present study considered only deaths in patients who received care in a hospice or long-term care facilities (including care homes and nursing homes). The number of beds available and patients admitted to HC facilities and the number of beds available in long-term care facilities during the study's period are presented in Supplementary Materials (Supplementary Table S1).

In Italy, hospice care is provided by the National Healthcare System (“Sistema Sanitario Nazionale” or SSN). The “Sistema Sanitario Nazionale” fully covers the costs of care. The number of palliative care units has continuously expanded since the mid-2000s and provides advanced home care, hospice, and consulting services. For this study, only hospice care is considered.

Long-term care facilities in Italy cover care homes and nursing homes. Care homes (“*Case di Riposo*”) are assisted living facilities for older adults who are partially self-sufficient and present primarily social needs. Nursing homes provide residential healthcare and social support to individuals with different clinical conditions including chronic patients who are not autonomous (i.e., those with dementia or disabilities).

### COVID-19 waves in Italy in 2020

Italy was the first nation with widespread population involvement in COVID-19, with high mortality in the spring of 2020, followed by a summer with relatively low infection incidence after easing the tight lockdown that was in effect from March 8 through May 2, 2020 [7]. The second wave arrived in late August and peaked in autumn 2020 [14]. Given the epidemiology of the COVID-19 pandemic in the country and the occurrence of two epidemic waves in 2020, for the present study, we present the distribution of death and causes of death that occurred in the country in the whole of 2020 and separately in the periods January–June and July–December.

## Results

**Fig. 1:**
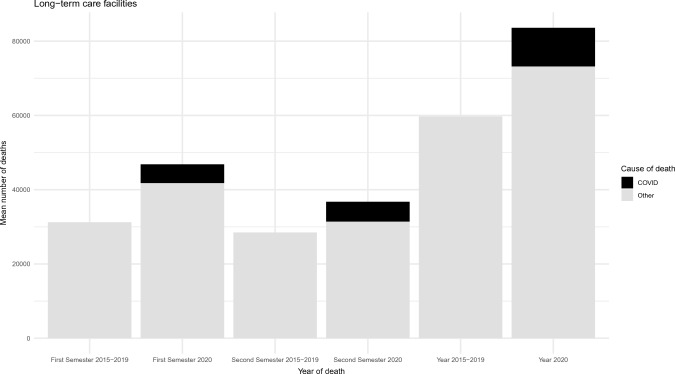
Mean number of deaths occurred in 2015–2019 and 2020 in Long-term care facilities. The black part of the columns represents deaths due to COVID-19

**Fig. 2:**
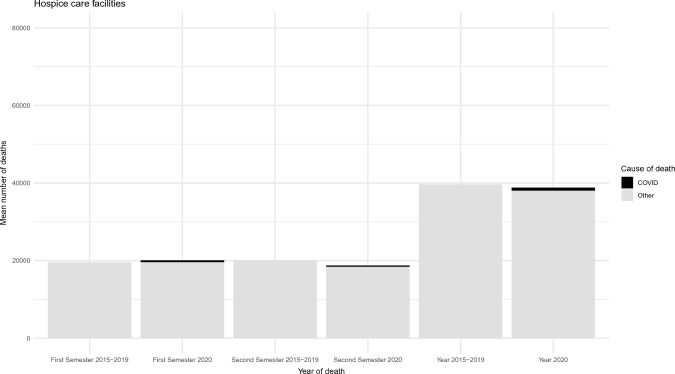
Mean number of deaths occurred in 2015–2019 and 2020 in Hospice care facilities. The black part of the columns represents deaths due to COVID-19

The mean number of deaths from all causes for 2015–2019 compared to 2020 is shown in Fig. 1 for hospice care facilities and in Fig. 2 for long-term care facilities. COVID-19 deaths are highlighted in black in the 2020 columns.

The figures show an increment in the total number of deaths in 2020 compared to the previous five years for long-term care facilities (83,062 vs. 59,200 deaths), consistent in both semesters analyzed. Conversely, a slight reduction was documented in the number of fatalities occurred in hospice care facilities (38,788 vs. 39,652), especially during the second semester of 2020. The number of deaths that occurred in long-term care facilities due to COVID-19 was 10,395 (12.5% of all deaths occurred in this setting in 2020). Deaths due to causes other than COVID-19 also increased in 2020 as compared with the 2015–2019 period (+ 16% deaths). COVID-19 seemed to have a minimal impact on deaths in hospice care facilities, with only 790 deaths due to COVID-19 (2% of all deaths occurred in this setting in 2020).

Table 1 shows sex distribution and mean age of deaths that occurred in hospice and long-term care facilities during the study periods. Patients who died in long-term care facilities were more likely females and older than those in hospice care facilities. Compared with the deaths registered during 2015–2019, patients who died in 2020 were more likely females and had an older age in both setting analyzed.

**Table 1:** Sex distribution and mean age of deaths occurred in hospice and long-term care facilities in the study periods

	Hospice care facilities		Long-term care facilities	
	% Males (*Mean Age*)	% Females (*Mean Age*)	% Males (*Mean Age*)	% Females (*Mean Age*)
First Semester				
2020	52.2% (74.3 y/o)	47.8% (75.9 y/o)	31.5% (84.2 y/o)	68.5% (88.8 y/o)
2015–2019	53.4% (73.7 y/o)	46.6% (75.3 y/o)	31.6% (83.8 y/o)	68.4% (88.4 y/o)
Second semester				
2020	51.8% (74.6 y/o)	48.2% (76.6 y/o)	30.8% (83.8 y/o)	69.2% (88.6 y/o)
2015–2019	53% (73.9 y/o)	47% (75.3 y/o	32.4% (83.8 y/o)	67.6% (88.2 y/o)

Finally, a comparison between the mean number of cause-specific deaths that occurred in the period 2015–2019 and the year 2020 is presented in Table 2. Data for hospice and long-term care facilities are shown according to the first and second semester periods and all-year data. In hospice care facilities, cancer was the most prevalent cause, accounting for 83% of all deaths in 2015–2019 and 77% in 2020. As already shown in Fig. 1, COVID-19 deaths were limited in this setting. In long-term care facilities, distribution of causes of death was more heterogeneous than in HC facilities. Cardiovascular diseases (25,565 deaths, 43% of all deaths), cancer (7,348 deaths, 12.4%), and psychotic and behavioral disorders (6,491 deaths, 11%) represented the most common causes of death during the period 2015–2019. Cardiovascular diseases were still the most common cause of death in this setting in 2020 (29,641 deaths, 35% of all deaths), followed by COVID-19 (10,395 deaths, 12.5%), and psychotic and behavioral disorders (8412 deaths, 10% of all deaths). With the only exception of cancer (-6.7%), the number of deaths for all other causes increased in 2020 as compared to 2015–2019. This association was consistent in both analyzed semesters, except for deaths due to respiratory diseases, which increased by 85% in the first semester and declined by 4.4% during the second semester in long-term care facilities.

**Table 2:** Causes of death in hospice care and long-term care facilities according to the first and second semester periods and all-year data (2020 vs 2015–2019)

	Hospice care facilities			Long-term care facilities			Hospice care facilities			Long-term care facilities			Hospice care facilities			Long-term care facilities		
	First Semester			First Semester			Second Semester			Second Semester			All years			All years		
Cause of Death	2020	2015–2019	VAR %	2020	2015–2019	VAR %	2020	2015–2019	VAR %	2020	2015–2019	VAR %	2020	2015–2019	VAR %	2020	2015–2019	VAR %
COVID-19	458	***	***	5054	***	***	332	***	***	5341	***	***	790	***	***	10395	***	***
Cancer	15653	16242	− 3.6	3732	3662	1.9	14209	16826	− 15.5	3124	3686	− 15.2	29862	33068	− 9.7	6856	7348	− 6.7
Cardiovascular disease	1609	1397	15.2	16246	13444	20.8	1556	1333	16.7	13,395	12,121	10.5	3165	2730	15.9	29641	25565	16
Psychotic and behavioral disorders	292	208	40.5	4829	3356	43.9	404	242	66.8	3583	3135	14.3	696	450	54.7	8412	6491	30
Respiratory diseases	444	322	37.9	4743	2564	85	396	284	39.2	1904	1992	− 4.4	840	606	38.5	6647	4556	45.8
Blood hematopoietic diseases and immune systems disorders	42	33	26.5	242	149	62.2	45	33	38	184	135	35.7	87	66	32.2	426	285	49.6
Endocrine, nutritional, and metabolic disorders	151	143	5.3	2096	1442	45.3	157	142	10.7	1768	1374	28.7	308	285	8	3864	2816	37.2
Central nervous system and sensory organs diseases	493	420	17.5	4065	2720	49.5	584	448	30.3	2973	2648	12.3	1077	868	24.1	7038	5368	31
Gastrointestinal diseases	264	267	− 1.2	561	464	21	297	261	13.6	483	460	5	561	529	6.1	1044	924	13
Skin and subcutaneous diseases	14	10	40	79	68	16.5	16	10	56.9	88	70	26.4	30	20	48.5	167	137	21.5
Musculo-skeleton and connective tissue diseases	40	26	52.7	285	198	43.7	24	29	− 16.7	219	203	8.1	64	55	16.3	504	401	25.7
Urinary tract diseases	160	119	34	723	509	42	147	116	26.3	645	482	33.8	307	236	30.2	1368	991	38
Trauma or poisoning	128	109	17.4	826	700	17.9	147	102	44.4	742	681	68.6	275	211	30.5	1568	1381	13.5
Infectious diseases	158	161	− 2	517	330	56.7	174	162	7.7	303	291	4.3	332	323	2.8	820	621	32.1
Other	149	106	40.3	2389	1175	103.4	245	99	147	1923	1141	9	394	205	91.2	4312	2316	86.22

## Discussion

We conducted a descriptive study to assess case-specific mortality in long-term care and hospice care facilities during the year 2020 in comparison to the previous five-year time (2015–2019), with a specific focus on COVID-19. We have underlined that COVID-19 had a relevant impact on deaths that occurred in LTCF, while hospice care facilities were less significantly affected by this condition. Residents who died during 2020 in long-term care facilities were on average older than those in hospice facilities and, more often, females. In Italy, independently of the study settings, an excess mortality of 108,178 deaths during the whole of 2020 compared to the previous five-year time was registered [25]. Based on the data we presented, we can estimate that 22% of this excess mortality can be explained by deaths that have occurred in long-term care facilities. This excess mortality can be attributed to COVID-19-related deaths as a primary cause, but also to under-treatment of other diseases as well as social isolation that might have increased depressive symptoms and delirium, both conditions associated with adverse outcomes, often leading to malnutrition, being bed-bound, and eventually death [26].

Especially during the first wave of the pandemic, long-term care facilities have been disproportionally affected by COVID-19, accounting for almost 50% of COVID-19-related deaths [27], and frail and vulnerable residents were not provided appropriate physical and psychosocial support, as underlined by Heckman and colleagues [28]. Residents of long-term care facilities with COVID-19 or other acute conditions, could often not be transferred to acute care hospitals, overwhelmed by COVID-19 patients. The increased number of deaths in 2020 cannot entirely be attributed to COVID-19 but also to several other underdiagnosed and undertreated conditions. Furthermore, during the initial phase of the epidemic, healthcare resources were often relocated to acute facilities to assist patients with COVID-19, and appointments or visits for chronic conditions were repeatedly postponed. Even the management of acute conditions different from COVID-19 was often delayed. The lack of PPE and low rate of SARS-CoV-2 testing made infection control challenging and diagnosis of COVID-19 uncertain or based on clinical symptoms, favoring the spreading of the virus within long-term care facilities. During the second semester of 2020, thanks to extra-governmental funds and increased availability of PPE, many long-term care facilities were transformed into COVID-19 facilities with the purpose of caring for older COVID-19 patients discharged from acute hospitals or promptly managing residents of the facilities whom SARS-CoV-2 infected. This factor can further explain the high number of deaths occurring in long-term care facilities.

Noticeably a substantial increment of deaths that occurred in long-term care facilities due to respiratory diseases was observed in the first semester of 2020. This finding can be due to the lack of SARS-CoV-2 diagnostic tests in the first period of the pandemic. Therefore, many patients may have been classified as dying by respiratory diseases (i.e., pneumonia) rather than by COVID-19.

The response to the pandemic in hospice care facilities was very limited, even if efforts to standardize palliative care for COVID-19 patients were made on a national scale. Hospice care facilities rarely activated an admission path for patients positive for SARS-CoV-2 infection. Italian hospices continued to register mainly cancer-related deaths while COVID-19-related deaths were extremely low. A survey conducted in the U.S. on hospice agencies underlined how healthcare professionals found that inadequate supplies of PPE had an essential impact on the delivery of care during the first year of the pandemic especially, as well as the reduced number of personnel in hospices insufficient to respond to the increase in family bereavement needs efficiently. More importantly, almost one-third of respondents reported adverse effects on patient outcomes, such as inadequate symptom management and negative psychosocial effects which can be explained by shorter visitations of healthcare providers and family members to reduce probabilities of contact [29]. The number of deaths due to cancer was substantially reduced in hospice facilities in 2020. This could relate the fact that cancer patients with terminal disease often remained hospitalized if affected by COVID-19 and were not transferred to hospice care facilities. In addition, community-dwelling patients with cancer may have experienced difficulties in accessing healthcare services, including palliative care, or may have avoided contact with healthcare services due to increased risk and fear of contracting COVID-19.

## Conclusions and implications

In conclusion, the data we have presented describe the scenario experienced in two different types of residential care facilities in Italy, during the first year of COVID-19 Pandemic. These data can help understand the strategies adopted in the first epidemic phase, when the National Healthcare System was unprepared to face the challenges posed by COVID-19 and to plan strategies to limit the impact of future epidemics.

## Supplementary Information

Below is the link to the electronic supplementary material.

Supplementary file1 (DOCX 14 KB)

## Data Availability

The data used for this work are public and available from the website of the Italian National Institute of Statistics (ISTAT).
