# Supplementary material for: Causes of deaths in long-term care and hospice care facilities during the first year of COVID-19 pandemic: a snapshot of Italy during 2020
Source: Aging Clin Exp Res. 2023 May 12;35(6):1385–92. doi: 10.1007/s40520-023-02426-7 (PMC10175910; doi:10.1007/s40520-023-02426-7)
Supplement: Supplementary file 1 — Supplementary file1 (DOCX 14 KB) [file 40520_2023_2426_MOESM1_ESM.docx]

**Supplementary Materials**

**Supplementary Table S1.** Number of beds and patients admitted per year in hospice and number of beds in long-term care facilities in Italy (year 2015-2020). Residential care includes nursing home, board and care homes and assisted living facilities.

| Years | 2015 | 2016 | 2017 | 2018 | 2019 | 2020 |
| --- | --- | --- | --- | --- | --- | --- |
| Hospice | | | | | | |
| Beds | NA | 2,940 | 3,099 | 3,138 | 3,382 | 3,236 |
| Patients admitted per year | NA | 44,388 | 45,799 | 47,208 | 50,229 | 44,387 |
| Long-term care facilities | | | | | | |
| Beds | 390,689 | 412,971 | 412,518 | 420,329 | 416,324 | 411,992 |

Data are from:

“Le strutture residenziali socio-assistenziali e socio-sanitarie al 31 dicembre 2020”. Istat – Nov 22, 2022. Available at https://www.istat.it/it/files//2022/11/REPORT_PRESIDI_SOCIO-ASSISTENZIALI_2020.pdf (accessed on Nov 23, 2022)

Italian Ministry of Health. Annuario Statistico del Servizio Sanitario Nazionale Assetto organizzativo, attività e fattori produttivi del SSN. Years 2016, 2017, 2018, 2019, 2020
